# High Prevalence and Gender-Related Differences of Gastrointestinal Manifestations in a Cohort of DM1 Patients: A Perspective, Cross-Sectional Study

**DOI:** 10.3389/fneur.2020.00394

**Published:** 2020-06-12

**Authors:** Alessia Perna, Daria Maccora, Salvatore Rossi, Tommaso Filippo Nicoletti, Maria Assunta Zocco, Vittorio Riso, Anna Modoni, Antonio Petrucci, Venanzio Valenza, Antonio Grieco, Luca Miele, Gabriella Silvestri

**Affiliations:** ^1^Institute of Neurology, Università Cattolica del Sacro Cuore, Rome, Italy; ^2^Department of Image Diagnostics, Oncological Radiotherapy and Hematology Sciences, Fondazione Policlinico Universitario A. Gemelli IRCCS, Rome, Italy; ^3^Department of Internal Medicine, Gastroenterology and Hepatology, Fondazione Policlinico Agostino Gemelli IRCCS, Rome, Italy; ^4^UOC of Neurology, Area of Neuroscience, Fondazione Policlinico Universitario A. Gemelli IRCCS, Rome, Italy; ^5^Center for Neuromuscular and Neurological Rare Disease, S. Camillo Forlanini Hospital, Rome, Italy; ^6^Department of Gastroenterological, Endocrine-Metabolic and Nefro-Urological Sciences, Fondazione Policlinico Universitario A. Gemelli IRCCS, Rome, Italy

**Keywords:** myotonic dystrophy type 1, gastrointestinal symptoms, multisystem involvement, gender-related differences, gastrointestinal symptom rating scale, intestinal permeability

## Abstract

Myotonic dystrophy type 1 (DM1, MIM #160900), the most common muscular dystrophy among adults, is a multisystem disorder, which affects, besides the skeletal muscle, several other tissues and/or organs, including the gastrointestinal apparatus, with manifestations that frequently affect the quality of life of DM1 patients. So far, only few, mainly retrospective studies evaluated this specific topic in DM1, so we performed a perspective study, enrolling 61 DM1 patients who underwent an extensive diagnostic protocol, including administration of the Gastrointestinal Symptom Rating Scale (GSRS), a validated patient-reported questionnaire about GI symptoms, laboratory tests, liver US scan, and an intestinal permeability assay, in order to characterize frequency and assess correlations regarding specific gastrointestinal manifestations with demographic or other DM1-related features. Our results in our DM1 cohort confirm the high frequency of various gastrointestinal manifestations, with the most frequent being constipation (45.9%). γGT levels were pathologically increased in 65% of DM1 patients and GPT in 29.82%; liver ultrasound studies showed steatosis in 34.4% of patients. Significantly, 91.22% of DM1 patients showed signs of altered intestinal permeability at the specific assay. We documented a gender-related prevalence and severity of gastrointestinal manifestations in DM1 females compared to DM1 males, while males showed higher serum GPT and γGT levels than females. Correlation studies documented a direct correlation between severity of muscle weakness estimated by MIRS score and γGT and alkaline phosphatase levels, suggesting their potential use as biomarkers of muscle disease severity in DM1.

## Introduction

Myotonic dystrophy type 1 or Steinert's disease (DM1, MIM #160900) is the most common form of adult-onset muscular dystrophy, with an estimated prevalence of about 1:8000 among Caucasians; recently, an epidemiological study conducted in the Rome province estimated a total age-standardized prevalence of 9.65/100,000 for DM1 (1).

DM1 is an autosomal dominant, multisystem disorder, caused by the pathologic expansion of a polymorphic CTG triplet repeat in the 3′ non-coding region of *DMPK* gene on chromosome 19q13.3, which encodes for the DM protein kinase (MIM#605377). *DMPK* alleles from healthy subjects contain 5–35 CTG repeats, while DM1 patients carry alleles with expanded repeats ranging from 50 to more than 1,000 CTG repeats. The expanded *DMPK* allele shows both mitotic and intergenerational instability biased toward expansion, which explain the inter-individual variability due to tissue mosaicism and the phenomenon of anticipation during linear transmission, respectively (2). Many studies have shown that the length of the CTG expansion in peripheral leukocytes inversely correlates with the age of onset and directly with the severity of muscle weakness (3, 4).

The pathogenesis of DM1 is complex, with a pivotal role played by the toxic effect of the mutant *DMPK* pre-mRNAs containing the expanded CUG stretch, which would eventually disrupt the expression of other genes in various tissues by impairing the function of specific transcription factors regulating alternative splicing. As the *DMPK* mRNA is widely expressed in many tissues, this explains the variable multisystem involvement in DM1 patients, also affecting the central nervous system, the eye, the heart, the smooth muscle, and the endocrine system, with the related development of cognitive and behavioral deficits, premature cataracts, cardiac conduction abnormalities, endocrine dysfunctions, and gastrointestinal (GI) symptoms.

Regarding GI manifestations, these can be referred even by children or adolescents affected by DM1; the most common disturbances related to involvement of upper gastrointestinal tract include dysphagia, heartburn, emesis, dyspepsia, vomiting, coughing while eating, and regurgitation, whereas abdominal pain, bloating, constipation, changes in bowel habits, diarrhea, pseudo-obstruction, and dyschezia are the most common complaints related to the lower digestive tract (5, 6). A moderate increase of serum gamma GT levels is also found in most DM1 patients, and liver echo scan often shows signs of liver steatosis and/or gallbladder stones (7, 8).

So far, only few studies have specifically assessed GI features in DM1 patients, mainly based on a retrospective analysis of collected data (9, 10) or only by questionnaires (5, 11).

These considerations prompted us to perform a perspective study to assess in detail the spectrum of specific gastrointestinal features in a cohort of 61 patients with molecular diagnosis of DM1, in order to estimate the prevalence of individual GI manifestations and analyze their correlation with demographic (i.e., gender, age) or DM1-specific features (i.e., age at onset of muscle symptoms, severity of muscle phenotype, nCTG in leukocytes).

## Materials and Methods

The study design was made according to the Declaration of Helsinki and approved by the Ethical Committee of our Institution (Prot. 28458/19 ID: 2665). All patients gave their written informed consent to partecipate to the study.

### Patients

The study cohort includes 61 adult DM1 patients (57.4% males, mean age at examination 47.20 ± 13.85 years) consecutively enrolled from January to November 2019 at the Department of Neurology of our Institution; eight of them, diagnosed and in follw-up at the *S. Camillo* Forlanini Hospital were referred by their neurologist (dr *A. Petrucci*) to our Center for the study enrollment and the instrumental diagnostic protocol. Inclusion criteria were as follows: age >18 years, molecular diagnosis of DM1with estimation of the nCTG size in leukocytes, and capacity to give informed consent to participate to this study and to the collection of their demographic and diagnostic data for clinical research purposes. All enrolled patients, except one positive to hepatitis C antibodies determination, were negative to hepatitis B and C and to celiac disease serologic testing.

Collected data for the study included the following:

- demographic data (gender, age);
- age at DM1 symptoms onset;
- DM1 disease duration (in years);
- Muscular Impairment Rating Scale (MIRS) (12) score assessed at the time of study enrolment;
- mean (CTG)n value detected in leukocytes at diagnosis;
- body mass index (BMI) assessed by weight [kg]/height [m]^2^: BMI <18.49 kg/m^2^ = underweight; 18.5–24.99 kg/m^2^ = normal weight; 25–29.99 kg/m^2^ = overweight; >30 kg/m^2^ = obesity;
- presence of comorbidities, such as hypertension, liver steatosis, diabetes type II;
- current medical therapies.

The GI diagnostic assessment included the following:

- administration of a validated self-reported questionnaire: presence and severity of GI symptoms were assessed by a limited version of the Gastrointestinal Symptom Rating Scale (GSRS) simplified in order to increase the compliance of DM1 patients, and concerning only five main symptom clusters (gastroesophageal reflux disease—GERD—item 3, abdominal discomfort—item 1, abdominal pain—item 4, constipation—item 10, and diarrhea—item 11 questionnaire (13, 14);
- laboratory tests, including serum fasting glucose (normal values < 110 mg/dl), glutamate pyruvate transaminase (GPT)/alanine aminotransferase (ALT) (normal values < 45 UI/L), gamma glutamyl transferase (γGT) (normal values < 36 UI/L), alkaline phosphatase (normal values < 104 UI/L), amylase (normal values < 106 UI/L), creatinine (normal values 0.67–1.17 mg/dl), total cholesterol (normal values < 200 mg/dl), triglycerides (normal values < 170 mg/dl), vitamin D levels (normal values 31–100 ng/mL), erythrocyte sedimentation rate (ESR) (normal values < 30 mm/h);
- liver ultrasound (US) scan: this test was performed after at least 6 h fasting with the patient in the supine decubitus position and the right arm in maximal abduction; the probe was placed in the right intercostal space that provides the best view of the right liver lobe. A B-mode scanning was performed to assess the presence and degree of liver steatosis and to select the most appropriate area of the right lobe (at least 5 cm in diameter) free of large vessels, and 2 cm below the liver capsule;
- intestinal permeability assay: for this test, after an overnight fast, patients were given to drink 0.37 MBq of ^51^Cr-EDTA (chromium-51 labeled ethylenediamine tetraacetic acid) (Amersham Health, England) in 10 ml of water. A sample corresponding to 1/50 of the total amount (0.1 ml) was collected and used as the standard sample. After drinking the parafarmaceutical, patients were asked to collect their 24-hour urine. Then a 3 ml patient's sample from the urine collection and the reference standard sample were both measured by gamma counter (LKB-Wallac 1282 Compugamma, Turku, Finland). To assess urinary excretion of ^51^Cr-EDTA, that was calculated using the following formula: (Mean Urinary Counts × Urinary Volume) × (Standard counts × 50)^−1^; the result was expressed as percentage of administered dose and considered indicative of altered intestinal permeability when the calculated percentage value was >3% (15, 16). ^51^Cr-EDTA is normally used for the evaluation of the glomerular filtration rate after an intravenous injection, in view of its glomerular extraction fraction of 1.0 ml/min/1.73 m^2^ and renal excretion time of 5 min. According to its high molecular weight, if administered orally, the intestinal ^51^Cr-EDTA absorption is minimal in healthy subjects (about 1–3%). On the contrary, in cases of increased transmural permeability due to any damage of the intestinal barrier, the amount of ^51^Cr-EDTA passing into the bloodstream increases proportionally with the extent and severity of the damage, followed by a rapid renal clearance (17).

### Statistical Analysis

Statistical analysis of data was performed by SPSS (Statistical Package for Social Science) version 24.0 to obtain descriptive statistics concerning demographic, clinical, molecular, and diagnostic data. To assess the presence of any associations between specific GI or other DM1 features with gender, we used the Mann–Whitney *U* test and the Fisher's two-tailed exact test to compare numerical and nominal variables between the two groups, respectively. In addition, Spearman correlation test was performed between numerical variables in pairs, to assess the presence of correlations between either (CTG)n, MIRS or BMI score with other numerical variables assessed in the cohort, including demographic characteristics (age at examination, age at onset, disease duration in years), serum laboratory tests (fasting glucose, GPT, GGT, alkaline phosphatase, amylase, creatinine, cholesterol, triglycerides, vitamin D, and ESR), and diagnostic tests (total IP expressed as % values) (Supplementary Table 1).

## Results

Overall (Table 1), our cohort included 35 male and 26 female adult DM1 patients. Mean age at onset was 32.34 ± 15.28 years, with a mean duration of disease of 14.85 ± 9.37 years. Mean MIRS score was 2.89 ± 0.84 (range 2–5), and (CTG)n size was 466.17 ± 269.32 (range 78–1200).

**Table 1:** Demographic, molecular and clinical characteristics of the cohort of study.

	**Count (%)**	**Mean (SD)**	**n**
Male	35 (57.4%)		61
Age at disease onset		32.34 (15.28)	61
Age at examination		47.20 (13.85)	61
Disease duration		14.85 (9.37)	61
BMI		24.21 (4.78)	61
BMI ≥ 30	8 (13.1%)		
(CTG)n		466.17 (269.32)	53
MIRS score		2.89 (0.84)	61
Comorbidities			61
Hypertension	11 (18%)		
Steatosis	21 (34.4%)		
Diabetes type II	6 (9.8%)		
Cholelithiasis	19 (31.1%)		
Cholecistectomy	12 (19.7%)		
Hepatitis C	1 (1.6%)		
GI symptoms	44 (72%)		61
GERD	24 (39.3%)		
GERD (GSRS score)		2.16 (1.69)	
Abdominal discomfort	27 (44.3%)		
Abdominal discomfort (GSRS score)		2.31 (1.80)	
Abdominal pain	20 (32.8%)		
Abdominal pain (GSRS score)		1.95 (1.62)	
Constipation	28 (45.9%)		
Constipation (GSRS score)		2.47 (1.83)	
Diarrhea	24 (39.3%)		
Diarrhea (GSRS score)		1.89 (1.38)	
Constipation/Diarrhea	15 (24.6%)		
Medications			61
Mexiletine	14 (23%)		
Anti-hypertensives	11 (18%)		
Aspirin	11 (18%)		
Insulin	3 (4.9%)		
Metformin	5 (8.2%)		
Ursodeoxycholic acid	5 (8.2%)		
Hypolipidemic drugs (ezetimibe or omega 3-fatty acids)	14 (23%)		
Anti-constipation medicines	7 (11.5%)		
PPIs	21 (34.4%)		
Probiotics	4 (6.6%)		
Vitamine D supplements	30 (49.2%)		
Blood tests			
Glucose		91.8 (26.69)	61
Impaired glucose tolerance (>110 UI/l)	7 (11.5%)		61
GPT		38.46 (24.77)	57
GPT out of range (>45 UI/l)	17 (29.82%)		57
GGT		88.22 (101.71)	60
GGT out of range (>36 UI/l)	39 (65%)		60
Alkaline phosphatase		80.81 (31.66)	54
Amylase		58.53 (29.97)	45
Total cholesterol		188.22 (40.62)	59
Hypercholesterolemia (>200 mg/dl)	21 (35.59%)		59
Triglycerides		152.41 (71.14)	59
Vitamine D		24.75 (13.05)	48
Vitamine D below the normal range (<30 UI/l)	32 (66.67%)		48
Creatinine		0.75 (0.25)	59
Erythrocyte Sedimentation Rate		14.39 (17.33)	46
Intestinal permeability (IP)			
Total IP		6.71 (4.57)	57
Altered	52 (91.22%)		57

*SD, standard deviation; BMI, Body Mass Index; MIRS, muscular impairment rating scale; GI, gastrointestinal; GERD, gastroesophageal reflux disease; GSRS, Gastrointestinal Symptom Rating Scale; NAFLD, non-alcoholic fatty liver disease; PPI, proton pump inhibitor; IP, intestinal permeability*.

Administration of the GSRS questionnaire documented that 72% of DM1 patients referred at least one GI symptom: the most common was constipation (45.9%), followed by abdominal discomfort (44.3%), GERD (39.3%), diarrhea (39.3%), abdominal pain (32.8%), and alternating constipation/diarrhea (24.6%). GSRS scores, indicating the severity of GI symptoms, were the highest for constipation (2.47 ± 1.83), followed by abdominal discomfort (2.31 ± 1.80), GERD (2.16 ± 1.69), abdominal pain (1.95 ± 1.62), and diarrhea (1.89 ± 1.38).

Regarding laboratory tests, γGT levels were pathologically increased (mean value 88.22 ± 101.71 UI/L) in 65% of DM1 patients, alkaline phosphatase (mean 80.81 ± 31.66 UI/L) in 19.7% of DM1 patients, and GPT in 29.82% of the patients; amylase was out of normal range in only 3 patients. Mean value for triglycerides was 152.41 ± 71.14 mg/dl; elevated total cholesterol (mean 187.39 ± 40.62 mg/dl) was found in 35.59% of patients.

Liver US showed steatosis in 34.4% of patients, and 31.1% of patients had cholelithiasis. Overall, 19.7% had undergone cholecystectomy for gallbladder stones. Only 8.2% of all DM1 patients took ursodeoxycholic acid. The DM1 patient positive for hepatitis C virus did not show any structural liver involvement at US studies.

Of note, most of our DM1 patients (91.22%) showed signs of altered intestinal permeability at the IP test.

Regarding comorbidities, 18% of patients suffered from hypertension, controlled by anti-hypertensive drugs in all of them. About 13% of our DM1 cohort was frankly obese (BMI > 30). 9.8% of patients suffered from type II diabetes, while reduced vitamin D was detected in about two thirds of patients.

Regarding pharmacological treatment of GI symptoms, 34.4% of patients took proton pump inhibitors (PPI); 11.5%, anti-constipation medicines; and 6.6%, long-term probiotics. Moreover, 23% of them assumed hypolipidemic drugs (either ezetimibe or omega 3-fatty acids), about 50% of patients took vitamin D oral supplements, 8.2% patients were in treatment with metformin, and 4.9% also required subcutaneous insulin.

Statistical analysis (Table 2) documented significant gender-related differences regarding frequency of GI complains: DM1 females (88.5%) referred at least one GI symptom more frequently compared to DM1 males (60%), with constipation being their main complain (65.4 vs. 31.4%), followed by abdominal discomfort (61.5 vs. 31.4%), and alternating constipation/diarrhea (38.5 vs. 14.3%). Also, the severity of GI symptoms by GSRS score was more severe in DM1 females than males: constipation (3.04 ± 1.82 vs. 2.06 ± 1.75) resulted the most severe, followed by abdominal discomfort (2.85 ± 1.80 vs. 1.91 ± 1.72), and abdominal pain (2.53 ± 1.90 vs. 1.51 ± 1.22). On the other hand, GERD and diarrhea resulted in having similar frequency and are mild in both DM1 groups.

**Table 2:** Statistical analysis for gender-related issues.

	**Male** **=** **35 (57.38%)**			**Female** **=** **26 (42.62%)**			**FET**	**MWUT**
	**Count (%)**	**Mean (SD)**	***n***	**Count (%)**	**Mean (SD)**	***n***		
Age at disease onset		30.54 (13.92)	35		34.77 (16.91)	26		NS
Age at examination		47.03 (11.50)	35		47.42 (16.74)	26		NS
Disease duration		16.49 (9.22)	35		12.65 (9.29)	26		NS
BMI		24.94 (5.01)	35		23.23 (4.35)	26		NS
BMI≥30	6 (17.1%)		35	2 (7.7%)		26	NS	
(CTG)n		427.87 (260.17)	31		506.05 (282.94)	22		NS
MIRS score		2.94 (0.80)	35		2.81 (0.90)	26		NS
Comorbidities								
Hypertension	6 (17.1%)			5 (19.2%)			NS	
Steatosis	14 (40.0%)		35	7 (26.9%)		26	NS	
Diabetes type II	5 (14.3%)		35	1 (3.8%)		26	NS	
Cholelithiasis	9 (25.7%)		35	10 (38.5%)		26	NS	
Cholecistectomy	7 (20.0%)		35	5 (19.2%)		26	NS	
Hepatitis C	0 (0%)		35	1 (3.8%)		26	NS	
≥1 GI symptoms	21 (60.0%)		35	23 (88.5%)		26	**0.020**	
GERD	14 (40%)		35	10 (38.5%)		26	NS	
GERD (GSRS score)		2.20 (1.71)			2.12 (1.70)			NS
Abdominal discomfort	11 (31.4%)		35	16 (61.5%)		26	**0.036**	
Abdominal discomfort (GSRS score)		1.91 (1.72)			2.85 (1.80)			**0.017**
Abdominal pain	8 (22.9%)		35	12 (46.2%)		26	NS	
Abdominal pain (GSRS score)		1.51 (1.22)			2.53 (1.90)			**0.021**
Constipation	11 (31.4%)		35	17 (65.4%)		26	**0.011**	
Constipation (GSRS score)		2.06 (1.75)			3.04 (1.82)			**0.020**
Diarrhea	11 (31.4%)		35	13 (50%)		26	NS	
Diarrhea (GSRS score)		1.69 (1.16)			2.15 (1.62)			NS
Alternating constipation/diarrhea	5 (14.3%)	1.69 (1.16)	35	10 (38.5%)		26	**0.039**	
Medications								
Mexiletine	9 (25.7%)		35	5 (19.2%)		26	NS	
Anti-hypertensives	6 (17.1%)		35	5 (19.2%)		26	NS	
Aspirin	6 (17.1%)		35	5 (19.2%)		26	NS	
Insulin	2 (5.7%)		35	1 (3.8%)		26	NS	
Metformin	2 (5.7%)		35	3 (11.5%)		26	NS	
Ursodeoxycholic acid	5 (14.3%)		35	0 (0%)		26	NS	
Hypolipidemic drugs	9 (25.7%)		35	5 (19.2%)		26	NS	
Anti-constipation medicines	4 (11.4%)		35	3 (11.5 %)		26	NS	
PPIs	13 (37.1%)		35	8 (30.8%)		26	NS	
Probiotics	3 (8.6%)		35	1 (3.8%)		26	NS	
Vitamine D supplements	19 (54.3%)		35	11 (42.3%)		26	NS	
Blood tests								
Glucose		92.57 (29.82)	35		90.77 (22.34)	26		NS
Glucose out of range (>110 UI/l)								
GPT		43.94 (26.45)	33		30.92 (20.46)	24		**0.006**
GPT out of range (>45 UI/l)	13 (60.6%)		33	4 (16.7%)		24	NS	
GGT		103.23 (89.71)	35		67.20 (115.08)	25		**0.002**
GGT out of range (>36 UI/l)	28 (80%)		35		11 (44%)	25	**0.006**	
Alkaline phosphatase		76.70 (26.60)	33		87.29 (38.11)	21		NS
Amylase		60.71 (34.41)	28		54.94 (21.22)	17		NS
Total cholesterol		186.20 (43.23)	35		191.17 (37.19)	24		NS
Triglycerides		161.31 (76.66)	35		139.42 (61.48)	24		NS
Vitamine D		24.65 (12.28)	28		24.89 (14.39)	20		NS
Vitamine D below the normal range (<30 UI/l)	19 (67.9%)		28	13 (65.0%)		20	NS	
Creatinine		0.84 (0.25)	34		0.63 (0.19)	25		**0.001**
Erythrocyte Sedimentation Rate		10.62 (10.77)	26		19.30 (22.67)	20		NS
Intestinal permeability (IP)								
Total IP		6.94 (5.02)	33		6.40 (3.97)	24		NS
Altered	31 (93.9%)		33		21 (87.5%)	24	NS	

*SD, standard deviation; BMI, Body Mass Index; MIRS, muscular impairment rating scale; GI, gastrointestinal; GERD, gastroesophageal reflux disease; GSRS, Gastrointestinal Symptom Rating Scale; NAFLD, non-alcoholic fatty liver disease; PPI, proton pump inhibitor; IP, intestinal permeability; FET, Fishers' exact test; MWUT, Mann-Whitney U Test. Bold indicates statistical significance of values*.

Conversely, serum GPT (43.94 ± 26.45 vs. 30.92 ± 20.46 UI/L) and γGT (103.23 ± 89.71 vs. 67.20 ± 115.08 UI/L) levels were both significantly higher in DM1 males vs. females, and γGT also resulted in being more frequently pathologically increased (80 vs. 44%). Finally, creatinine serum levels were normal in all patients, being also higher in males (0.84 vs. 0.63 mg/dl).

Correlation analysis by Spearman's test (Supplementary Table 1) showed that:

- (CTG)n inversely correlated only with age at onset of disease (*r* = −0.326; *p* = 0.017);
- MIRS score directly correlated with age at the examination (*r* = 0.401; *p* = 0.001), disease duration (*r* = 0.473; *p* ≤ 0.001), GGT (*r* = 0.390; *p* = 0.002), and alkaline phosphatase levels (*r* = 0.362; *p* = 0.007);
- BMI directly correlated with disease duration (*r* = 0.364; *p* = 0.004), GPT (*r* = 0.356; *p* = 0.007), and γGT levels (*r* = 0.316; *p* = 0.014).

## Discussion

The aim of this study was to assess in detail the prevalence and the characteristics of GI involvement in DM1 patients. To accomplish this, we performed a perspective study on a significant cohort of DM1 patients characterized about the occurrence of gastrointestinal involvement by an extensive diagnostic panel: this included subjective evaluation of the prevalence and severity of GI manifestation by administering a validated GI questionnaire, GSRS (13, 14), and objective assessment of GI manifestations by blood and instrumental tests, including US liver and a radionuclide-based assay for the intestinal permeability (15).

The results of our study confirm a very high prevalence of GI manifestations in DM1: indeed, 44/61 of our patients (72.1%) referred at least one GI symptom, including constipation (45.9%), referred GERD, and diarrhea (39.3%).

The prevalence of GI symptoms in our study is higher than that reported by Hilbert et al. (5), who assessed the prevalence of GI manifestations in a very large cohort of DM1 patients based on data obtained from the National Registry of Myotonic Dystrophy; indeed, in that study, 38% of DM1 patients referred GERD, 33% constipation, and only 1.3% diarrhea. However, our perspective study assessed the presence of GI symptoms using one questionnaire, GSRS, specifically validated, and certainly more sensitive to detect the presence of GI symptoms (16–19). Given the need of validated outcome measures to assess various systemic manifestations of DM1, we are planning to validate specifically for DM1 this limited version of Gastrointestinal Symptoms Rating Scale (GSRS), concerning the domains depicting constipation, diarrhea, abdominal pain, abdominal discomfort, GERD, as it resulted in an easy, quick, and sensitive instrument for the assessment of GI symptoms in DM1 patients. By this diagnostic tool, a significant percentage of our DM1 patients reported various GI symptoms, including abdominal discomfort (44.3%), abdominal pain (32.8%), and/or alternating constipation/diarrhea (24.6%). By using GSRS, we were also able to score the severity of GI symptoms, which showed higher values for constipation (2.47 ± 1.83), followed by abdominal discomfort (2.31 ± 1.80), GERD (2.16 ± 1.69), abdominal pain (1.95 ± 1.62), and diarrhea (1.89 ± 1.38).

Regarding diagnostic tests, confirming our previous observations (20), liver US documented a significant prevalence of steatosis (34.4%), and gallbladder stones (total 31.1%). The significant prevalence of such manifestations in DM1 is also highlighted by another study conducted on 31 DM1 patients (21) documenting the presence of liver steatosis with abnormal liver tests in 54% of cases.

Most strikingly, the majority of our DM1 patients (91.22%) showed an altered intestinal permeability. We performed the intestinal permeability test in DM1 patients, since a previous study (22) had shown that non-alcoholic fatty liver disease (NAFLD) was significantly associated with an increased gut permeability caused by disruption of intercellular tight junctions, which could therefore play an important role in the pathogenesis of hepatic fat deposition.

Although an abnormal IP occurred virtually in all our DM1 patients, liver US scan documented steatosis only in 34.4% of them, and these data might suggest that an increased IP might represent a very early GI manifestation in DM1, preceding the occurrence of liver steatosis.

Determinants of fatty liver degeneration in DM1 are not yet clarified, and so far, only one study (7) analyzing a cohort of 36 DM1 patients, documented a high prevalence of NAFLD (about 38% of patients) and its strong association with markers of insulin resistance and features of the metabolic syndrome. NAFLD is strongly associated with insulin resistance and type 2 diabetes (23), which in turn are associated with DM1 (24), being likely related to the aberrant expression of the insulin receptor documented in DM1 peripheral tissues (25–27). To clarify this issue, we are going to evaluate in a further study prevalence and predictors of NAFLD in our cohort of DM1 patients, in comparison to a non-DM1-NAFLD cohort. NAFLD is strongly associated with insulin resistance and type 2 diabetes (23): both these conditions are frequent features also in DM1 (24), being likely related to the aberrant expression of the insulin receptor in the peripheral tissues (25–27) and/or to the presence of other post-receptor signaling abnormalities in DM1 tissues (28).

It is recognized that an impaired motility of the GI smooth muscle, either caused by primary myofiber dysfunction or secondary to neural damage (11, 29), and with a possible concomitant contribution from endocrine dysfunctions (29), underlies the various GI disturbances affecting DM1 patients.

In this regard, the altered intestinal permeability documented in most of our DM1 patients might be consequent to the gut dysmotility, which could eventually cause a bacterial overgrowth (30) responsible for diarrhea or alternating alvus, while a delayed and reduced gallbladder contraction consequent to the toxic RNA effect occurring in its tissues (8) might predispose to gallstone formation.

Moreover, contractions of bile canaliculi and bile ductules (29) could be responsible for the elevation of γGT detected in DM1 patients (10, 31–33), that we confirmed also in our DM1 cohort (65% of cases). Also, our laboratory findings confirmed, in agreement with previous studies (29, 33–35), increased levels of serum alkaline phosphatase, GPT, triglycerides, and total cholesterol values in a significant percentage of DM1 patients (Table 1), although with some differences in the individual prevalence of such alterations possibly depend on different dietary habits.

Also, the prevalence of comorbidities such as hypertension, type 2 diabetes, and obesity was similar in our cohort to those reported by other studies (35).

In agreement with previous studies (5, 9, 10), our study confirms the presence of significant gender-related differences regarding frequency and severity of GI manifestations in DM1 (Table 2), with female patients showing a higher prevalence of GI symptoms, and males showing higher serum GPT and γGT levels. Such differences might be related to several factors, including hormonal status or sex differences in the gut microbiota composition (36), which of course deserve further studies. On the other hand, we did not find any gender-related differences for other metabolic parameters (Table 2).

This study also confirmed the high frequency of low serum vitamin D levels in DM1 patients originally described by Terracciano et al. (37) in this regard, the frequent intestinal permeability alterations in our cohort of DM1 patients might be the cause for an intestinal malabsorption of vitamin D, although another study suggests that in DM1 patients, low vitamin D levels could be due to reduced sun exposure due to gait impairment or to low intake of nutrients (38).

Besides the known correlations between nCTG and age at onset or MIRS and age or disease duration, statistical analysis interestingly documented a direct correlation between severity of muscle weakness estimated by MIRS score, γGT, and alkaline phosphatase levels, and these results would suggest that both these indexes might be considered if this correlation would be confirmed in a larger number of DM1 patients, as potential disease biomarkers in DM1 in view of future therapeutic trials.

Moreover, the correlation between BMI and γGT levels would support the pathogenic contribution of insulin resistance in the etiology of NAFLD in DM1.

The main limitation of this study concerns the relatively small study cohort, including 61 DM1 patients; nevertheless, their deep and homogeneous diagnostic characterization, particulrly considering that DM1 is a rare disease, represent, to our opinion, a strenght of this work. Another limitation consists in the the lack of diagnostic tests assessing the upper GI tract involvement; however, we previously documented via a retrospective analysis on 70 DM1 patients a high frequency of upper GI dysfunction tract by means of oropharyngoesophageal scintigraphy (OPES), which, in our experience, becomes an appropriate diagnostic test to assess and follow-up swallowing function in DM1 patients (20). Also, we did not assess in such cohort the presence of common lifestyle habits (smoke, alcohol intake) that may influence the presence of GI symptoms or liver steatosis yet we did not document a significant prevalence of such habits in a previous study cohort concerning cancer risk in DM1 (39).

In conclusion, we documented a high prevalence, with individual specific gender-related differences, of gastrointestinal manifestations in DM1. Thus, the gender of DM1 patients should be considered in the design of both medical management and clinical trials.

Further studies are certainly needed to fully clarify the complex pathophysiology of the gastrointestinal involvement in DM1, in order to define specific guidelines regarding diagnostic assessment and treatment of the various gastrointestinal disturbances in DM1 patients, as they often affect the quality of life of DM1 patients.

## Data Availability Statement

Beside the Supplementary Table, the raw data supporting the conclusions of this manuscript will be made available by the authors, without undue reservation, to any qualified researcher.

## Ethics Statement

The studies involving human participants were reviewed and approved by the Ethical Committee of Policlinico Gemelli IRCCS of Rome (Prot. 28458/19 ID: 2665). The patients/participants provided their written informed consent to participate in this study.

## Author Contributions

APer: participation to conceptual design, data collection and analysis, and manuscript writing. DM, TN, MZ, VR, AM, APet, and VV: diagnostic assessment and data collection and analysis. AG and LM: study design and critical revision of the manuscript for important intellectual content. SR: data statistical analysis and critical revision of the manuscript. GS: study concept and design, critical revision of the manuscript for important intellectual content, and manuscript editing.

## Conflict of Interest

The authors declare that the research was conducted in the absence of any commercial or financial relationships that could be construed as a potential conflict of interest.

## Abbreviations

DM1: myotonic dystrophy type 1
GI: gastrointestinal
OPES: oropharyngoesophageal scintigraphy
BMI: body mass index
MIRS: Muscular Impairment Rating Scale
GPT: glutamate pyruvate transaminase
ALT: alanine aminotransferase
γGT: gamma glutamyl transferase
ESR: erythrocyte sedimentation rate
US: ultrasound
^51^Cr-EDTA: chromium-51-labeled ethylenediamine tetraacetic acid
GSRS: Gastrointestinal Symptom Rating Scale
GERD: gastroesophageal reflux disease
NAFLD: non-alcoholic fatty liver disease
PPI: proton pump inhibitors.
