# Supplementary material for: High Prevalence and Gender-Related Differences of Gastrointestinal Manifestations in a Cohort of DM1 Patients: A Perspective, Cross-Sectional Study
Source: Front Neurol. 2020 Jun 12;11:394. doi: 10.3389/fneur.2020.00394 (PMC7303304; doi:10.3389/fneur.2020.00394)
Supplement: Supplementary file 1 [file Table_1.docx]

*Supplementary Table 1. Spearman correlations. Abbreviations: BMI, Body Mass Index; MIRS, muscular impairment rating scale; GPT, glutamate pyruvate transaminase; GGT, gamma glutamyl transferase; ESR, erythrocyte sedimentation rate; IP, intestinal permeability.*

|  | **(CTG)n** | | | **MIRS score** | | | **BMI** | | |
| --- | --- | --- | --- | --- | --- | --- | --- | --- | --- |
|  | **Correlation Coefficient** | **Sig. (2-tailed)** | **N** | **Correlation Coefficient** | **Sig. (2-tailed)** | **N** | **Correlation Coefficient** | **Sig. (2-tailed)** | **N** |
| **Age** | -0,216 | 0,12 | 53 | ***,401*** | ***0,001*** | ***61*** | 0,086 | 0,511 | 61 |
| **Disease onset** | ***-,326*** | ***0,017*** | ***53*** | 0,065 | 0,62 | 61 | -0,087 | 0,507 | 61 |
| **Disease duration** | 0,228 | 0,101 | 53 | ***,473*** | ***0*** | ***61*** | ***,364*** | ***0,004*** | ***61*** |
| **(CTG)n** | / | | | 0,224 | 0,106 | 53 | -0,004 | 0,979 | 53 |
| **MIRS score** | 0,224 | 0,106 | 53 | / | | | 0,134 | 0,302 | 61 |
| **BMI** | -0,004 | 0,979 | 53 | 0,134 | 0,302 | 61 | / | | |
| **Fasting glucose** | 0,137 | 0,327 | 53 | 0,115 | 0,378 | 61 | 0,075 | 0,565 | 61 |
| **GPT** | 0,098 | 0,499 | 50 | 0,095 | 0,481 | 57 | ***,356*** | ***0,007*** | ***57*** |
| **GGT** | 0,026 | 0,856 | 53 | ***,390*** | ***0,002*** | ***60*** | ***,316*** | ***0,014*** | ***60*** |
| **Alkaline phosphatase** | 0,243 | 0,096 | 48 | ***,362*** | ***0,007*** | ***54*** | 0,046 | 0,744 | 54 |
| **Creatinine** | -0,273 | 0,053 | 51 | -0,237 | 0,071 | 59 | 0,24 | 0,067 | 59 |
| **Cholesterol** | -0,003 | 0,985 | 52 | 0,036 | 0,784 | 59 | 0,007 | 0,961 | 59 |
| **Triglycerides** | -0,011 | 0,938 | 52 | 0,142 | 0,284 | 59 | 0,205 | 0,12 | 59 |
| **Vitamine D** | 0,054 | 0,734 | 42 | -0,081 | 0,584 | 48 | -0,039 | 0,795 | 48 |
| **ESR** | 0,183 | 0,252 | 41 | ***,428*** | ***0,003*** | ***46*** | 0,016 | 0,918 | 46 |
| **IP** | 0,024 | 0,87 | 50 | -0,057 | 0,675 | 57 | 0,115 | 0,396 | 57 |
